# Changes of b2-microglobulin and electrolytes in different stages of COPD and their value in evaluating prognosis

**DOI:** 10.5937/jomb0-50905

**Published:** 2024-11-16

**Authors:** Lin Wang, Rong Yi, Lanlan Wei, Jiali Xiong

**Affiliations:** 1 Central South University, Zhuzhou Hospital Affiliated to Xiangya School of Medicine, Department 2 of Respiratory and Critical Medicine, Zhuzhou, China

**Keywords:** b2-microglobulin, electrolyte, severity of COPD, b2-mikroglobulin, elektrolit, ozbiljnost HOBP

## Abstract

**Background:**

To investigate the changes of b2-microglobulin and electrolyte in different stages of chronic obstructive pulmonary disease (COPD) and the value of evaluating prognosis.

**Methods:**

A retrospective study was undertaken on 120 patients diagnosed with COPD and treated at our respiratory department between February 1, 2020, and January 31, 2023. These patients were classified into three groups based on the GOLD classification: mild (FEV1 > 81%), moderate (51% < FEV1 ≤ 80%), and severe (FEV1 ≤ 50%). As a control group, 40 healthy individuals who had routine examinations during the same period were selected. The COPD patients were then followed up and divided into a good prognosis group (n = 67) and a poor prognosis group (n = 53). The levels of b2-microglobulin and electrolytes were measured in patients with different stages of the disease and different prognoses. Kendall's tau-b and ordered logistic regression were employed to analyze how the changes in b2-microglobulin and electrolyte levels correlated with disease severity. Furthermore, the prognostic value of b2-microglobulin and electrolyte levels in COPD was assessed using an ROC curve.

**Results:**

In comparison to the control group, the severity of COPD patients displayed a notable increase in b2microglobulin levels, while there was a significant decrease in levels of calcium, chlorine, potassium, and sodium. Kendall's tau-b correlation coefficient analysis indicated a positive correlation between COPD severity and b2microglobulin, and a negative correlation between COPD severity and levels of calcium, chlorine, potassium, and sodium. Logistic regression analysis revealed that there was a positive correlation between disease severity and b2microglobulin, and a negative correlation between disease severity and levels of calcium, chlorine, potassium, and sodium. Furthermore, the poor prognosis group exhibited a significant increase in b2-microglobulin levels, alongside a significant decrease in levels of calcium, chlorine, potassium, and sodium compared to the good prognosis group (P < 0.05). ROC curve analysis demonstrated that a combined detection of b2-microglobulin, calcium, chlorine, potassium, and sodium yielded significantly higher area under the curve, sensitivity, and specificity values compared to single detection methods, highlighting its significant predictive value for COPD prognosis.

**Conclusions:**

Patients who presented with a more severe form of the disease exhibited elevated levels of b2microglobulin and reduced electrolyte levels. Prognostic accuracy was significantly enhanced when b2-microglobulin and electrolyte levels were analyzed together, offering a superior method for predicting patient outcomes.

## Abstract (sr)

**Uvod:**

Cilj je bio da se ispitaju promene b2-mikroglobulina i elektrolita u različitim stadijumima hronične opstruktivne plućne bolesti (HOBP) i vrednosti procene prognoze.

**Metode:**

Retrospektivna studija je sprovedena na 120 pacijenata sa dijagnozom HOBP i lečenih na našem respiratornom odeljenju u periodu od 1. februara 2020. do 31. januara 2023. Ovi pacijenti su klasifikovani u tri grupe na osnovu GOLD klasifikacije: blagi (FEV1 > 81%), umereno (51% < FEV1 ≤ 80%) i teško (FEV1 ≤ 50%). Kao kontrolnu grupu odabrano je 40 zdravih osoba koje su u istom periodu bile na rutinskim pregledima. Pacijenti sa HOBP su zatim praćeni i podeljeni u grupu sa dobrom prognozom (n = 67) i grupu sa lošom prognozom (n = 53). Nivoi b2-mikroglobulina i elektrolita su mereni kod pacijenata sa različitim stadijumima bolesti i različitim prognozama. Kendallova tau-b i naređena logistička regresija su korišćeni da se analizira kako su promene u b2-mikroglobulinu i nivoima elektrolita u korelaciji sa ozbiljnošću bolesti. Štaviše, prognostička vrednost b2-mikroglobulina i nivoa elektrolita u HOBP je procenjena korišćenjem ROC krive.

**Rezultati:**

U poređenju sa kontrolnom grupom, težina bolesnika sa HOBP pokazala je značajno povećanje nivoa b2-mikroglobulina, dok je došlo do značajnog smanjenja nivoa kalcijuma, hlora, kalijuma i natrijuma. Kendallova analiza koeficijenta tau-b korelacije pokazala je pozitivnu korelaciju između težine HOBP i b2-mikroglobulina, i negativnu korelaciju između težine HOBP i nivoa kalcijuma, hlora, kalijuma i natrijuma. Logistička regresiona analiza je otkrila da postoji pozitivna korelacija između težine bolesti i b2-mikroglobulina i negativna korelacija između težine bolesti i nivoa kalcijuma, hlora, kalijuma i natrijuma. Pored toga, grupa sa lošom prognozom pokazala je značajno povećanje nivoa b2-mikroglobulina, uz značajno smanjenje nivoa kalcijuma, hlora, kalijuma i natrijuma u poređenju sa grupom sa dobrom prognozom (P <0,05). Analiza ROC krive je pokazala da je kombinovana detekcija b2-mikroglobulina, kalcijuma, hlora, kalijuma i natrijuma dala značajno veću površinu ispod krive, osetljivost i vrednosti specifičnosti u poređenju sa pojedinačnim metodama detekcije, naglašavajući njegovu značajnu prediktivnu vrednost za prognozu HOBP.

**Zaključak:**

Pacijenti koji su imali teži oblik bolesti su ispoljili povišen nivo b2-mikroglobulina i smanjen nivo elektrolita. Prognostička tačnost je značajno poboljšana kada su b2-mikroglobulin i nivoi elektrolita analizirani zajedno, nudeći superiorni metod za predviđanje ishoda pacijenata.

## Introduction

Chronic obstructive pulmonary disease (COPD) is a leading cause of morbidity and mortality globally. It is characterized by persistent airflow limitation, reversible to some extent, along with chronic airway inflammation and emphysema. The World Health Organization has included COPD as one of the »four chronic diseases,« along with cardiovascular diseases, cancer, and diabetes. In China, the prevalence of COPD has been steadily increasing. Studies indicate that the prevalence rate of COPD in adults aged 20 and above is 8.6% [1]. This rate rises to 13.7% for individuals over 40 years old and exceeds 27% for those over 60 years old. The prevalence rate also exhibits a gender difference, with males being affected 2.2 times more than females. In China alone, the total number of COPD patients is nearing 100 million [2]. If left untreated, the chronic airway inflammation and recurrent infections associated with COPD can progress to bronchiectasis, causing further discomfort for patients [3]. Therefore, it is crucial to identify indicators that can assess changes and prognosis in COPD patients. One such indicator is β2-microglobulin, a small protein involved in various physiological processes, including immune response, inflammatory response, and cell proliferation. β2-microglobulin primarily undergoes filtration in the glomeruli followed by reabsorption in the proximal tubules. In individuals with COPD, the glomerular filtration rate increases as a result of the inflammatory response and oxidative stress, leading to elevated urinary excretion of β2-microglobulin [4]. Some studies have suggested that β2-microglobulin may enhance the susceptibility to age-related chronic neurodegenerative diseases by impairing the cognitive function and regenerative capacity of the hippocampus [5]. Electrolyte imbalances, such as hypokalemia and hypochloremia, are commonly observed in COPD patients. The chronic inflammation, hypoxia, respiratory acidosis, and other factors associated with COPD can disrupt the kidney’s ability to regulate electrolytes, thus contributing to electrolyte disorders. Moreover, due to the impact of lung function in COPD patients, hypoxia and metabolic abnormalities can also contribute to electrolyte imbalances. Severe electrolyte imbalances may impact disease progression and patient prognosis [6]. Therefore, in this study, patients were categorized into groups based on GOLD grade to investigate the expression of β2-microglobulin and electrolyte levels in individuals with different disease severity and prognosis. Furthermore, the prognostic value of β2-microglobulin and electrolyte levels in COPD patients was examined using ROC curve analysis, aiming to provide valuable insights for clinical disease assessment and prognosis evaluation.

## Materials and methods

### General information

From February 1, 2020 to January 31, 2023, a total of 120 COPD patients were retrospectively analyzed in our respiratory department. Inclusion criteria for participant selection were as follows: 1) patients were required to meet the diagnostic and treatment criteria for chronic obstructive pulmonary disease [7]; 2) patients had to exhibit significant cognitive impairment and demonstrate effective cooperation in the context of the study; moreover, they were expected to independently complete all relevant evaluations as required by the research; 3) patients were limited to an age not exceeding 80 years. Conversely, exclusion criteria were outlined as follows: 1) with major disease that results in a life expectancy of less than 1 year, such as a malignant tumor; 2) patients with severe hepatic and renal dysfunction were not considered eligible; 3) individuals with incomplete clinical data or whose disease had worsened were also excluded.

### Research methods

### Collection of serum samples

In the morning, the participants provided a 5 mL blood sample from the cubital vein while in a fasting state. The blood was then placed in an EDTA vacuum collection tube to prevent coagulation. To obtain the supernatant, a low temperature and high-speed centrifuge (EppendorfAG, model: 5425R) was used at a speed of 4500 r/min. The resulting supernatant was stored at -80 in a cryogenic refrigerator (TH-86-500-LA).

### Follow-up

COPD patients were divided into a good prognosis group of 67 cases and a poor prognosis group of 53 cases based on their disease remission or deterioration 28 days after admission. The group with good prognosis was considered based on the improvement of the patient’s condition, while the group with poor prognosis was considered based on the deterioration of the patient’s condition (such as increased white blood cell count and inflammatory cell count, respiratory frequency exceeding 20 times/min, changes in consciousness status, and elevated PaCO_2_) [8].

### Detection of serum indexes

The concentration of serum β2-microglobulin in patients with varying stages and prognosis was assessed using an automated biochemical analyzer (URIT-8036, Chengdu Yike Instruments and Equipment Co., Ltd., Chengdu, China). To determine the levels of serum β2-microglobulin in patients with different stages and prognosis, an electrolyte analyzer (HC-9886, Shenzhen Hangchuang Medical Equipment Co., Ltd., Shenzhen, China) was employed.

### Statistical analysis

The data analysis in this study was performed using the Statistic Package for Social Science (SPSS) 23.0 statistical software (IBM, Armonk, NY, USA). The patients were classified according to the GOLD classification into three groups: mild group (FEV1 > 81%), moderate group (n = 37), and severe group (FEV1 ≤ 50%) [9]. The measurement data, such as β2-microglobulin and electrolyte index, were examined using an independent sample t-test. To compare multiple groups, a single factor analysis of variance was utilized. The data related to sex and other counts were represented using (example (%)), and the comparison between groups was conducted using a χ^2^ test. Kendall’s tau-b and ordered logistic regression analysis were employed to investigate the relationship between changes in β2-microglobulin and electrolyte levels and disease severity. The prognostic value of β2-microglobulin and electrolyte levels in patients with COPD was assessed through ROC curve analysis. The observed differences were found to be statistically significant with a significance level of P < 0.05.

## Results

The mild group consisted of 34 males and 20 females, with an average age of (53.94 ± 6.01) years. Similarly, the moderate group had 20 males and 17 females, and the average age was (52.60 ± 6.46) years. In the severe group, there were 15 males and 14 females, with an average age of (53.00 ± 7.79) years. To establish a control group, 40 healthy subjects who underwent routine examination during the same period were selected, with an average age of (53.35 ± 5.52) years, including 25 males and 15 females. No statistically significant differences were observed in the general data between the patient groups and the control group (P > 0.05). Additionally, there were no statistically significant differences in age, sex, and other general data among the different patient groups (P > 0.05). The detailed data can be found in Table 1.

**Table 1:** General data analysis and comparison.

Group	N	Age years	Gender %		Smoking history %
			Male	Female	
Control group	40	53..27±6.96	25 62.50	15 37.50	13 32.50
Mild group	54	53.84±5.60	34 62.96	20 37.04	17 31.48
Moderate group	37	52.69±6.41	20 54.05	17 45.95	16 43.24
Severe group	29	53.09±7.24	15 51.72	14 48.28	15 51.72
*F*		0.25	1.555		4.231
*P*		0.864	0.670		0.238

### Analysis of β2-microglobulin and electrolyte levels in patients with different stages of COPD

**Figure 1:**
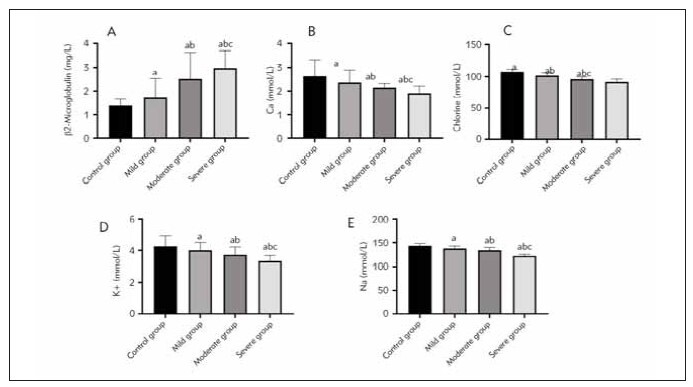
The comparison of β2-microglobulin and electrolyte levels in patients with 1COPD at different stages is presented. (A) depicts the juxtaposition of β2-microglobulin levels in the various groups. (B) showcases the contrast in calcium levels among the groups. (C) emphasizes the variability in chlorine levels across the groups. (D) highlights the divergen ce in potassium levels among the groups. Lastly, E demonstrates the disparities in sodium levels within all groups. It is worth noting that statistical analysis indicated significant differences (^a^P < 0.05) when compared to the control group, mild group (^b^P < 0.05), and moderate group (cP < 0.05).

In comparison to the control group, the mild group exhibited a significant increase in the level of β2-microglobulin, as well as a significant decrease in the levels of calcium, chlorine, potassium, and sodium (P < 0.05). Similarly, when comparing the moderate group to itself, there was a notable decrease in the levels of calcium, chlorine, potassium, and sodium (P < 0.05). However, when comparing the moderate group to the severe group, there was a significant increase in the level of β2-microglobulin in the severe group, accompanied by significant reductions in calcium, chlorine, potassium, and sodium (P < 0.05). The detailed data can be found in Table 2 and Figure 1.

**Table 2:** COPD comparison of β2-microglobulin and electrolyte levels in patients with different stages of disease.

Group	N	β2-microglobulin<br>(mg/L)	Calcium<br>(mmol/L)	Chlorine<br>(mmol/L)	Potassium<br>(mmol/L)	Sodium<br>(mmol/L)
Control group	40	1.40±0.27	2.64±0.69	107.84±3.77	4.25±0.68	142.70±5.52
Mild group	54	1.73±0.82^a^	2.38±0.52 a	101.86±4.73^a^	4.01±0.50^a^	137.33±4.87^a^
Moderate group	37	2.53±1.10^ab^	2.15±0.19 ab	96.23±3.58^ab^	3.72±0.49^ab^	133.52±5.72^ab^
Severe group	29	2.98±0.73^abc^	1.91±0.30 ^abc^	91.80±4.62^abc^	3.33±0.35^abc^	121.79±3.43^abc^
F		30.050	14.450	94.980	19.320	102.700
P		<0.001	<0.001	<0.001	<0.001	<0.001

### Kendall’s tau-b correlation analysis

According to Kendall’s tau-b correlation coefficient test, the severity of COPD is positively correlated with β2-microglobulin (Kendall’s tau-b=0.790, P<0.05), and negatively correlated with calcium, chloride, potassium, and sodium levels (Kendall’s taub=-0.751, -0.774, -0.757, -0.768, *P*<0.05). Table 3

**Table 3:** The relationship between the changes of β2-microglobulin and electrolyte levels and the severity of the disease.

Index	β	S.E. Wald	*P*	
β2-microglobulin	0.126	0.041	11.482	0.016
Calcium	-0.119	0.097	6.751	0.042
Chlorine	-0.136	0.115	17.540	0.007
Potassium	-0.179	0.173	10.576	0.020
Sodium	-0.063	0.120	8.667	0.037

### Sequential Logistics regression analysis

After adjusting for multiple confounding factors such as gender and age in multivariate analysis, β2-microglobulin, calcium, chloride, potassium, and sodium were used as variables in the logistic regression analysis. Regression analysis showed that β2-microglobulin had a significant positive correlation with the severity of the disease (P<0.05), while calcium, chloride, potassium, and sodium had a significant negative correlation with the severity of the disease (P<0.05).

### Analysis of β2-microglobulin and electrolyte levels in patients with different prognosis

**Figure 2:**
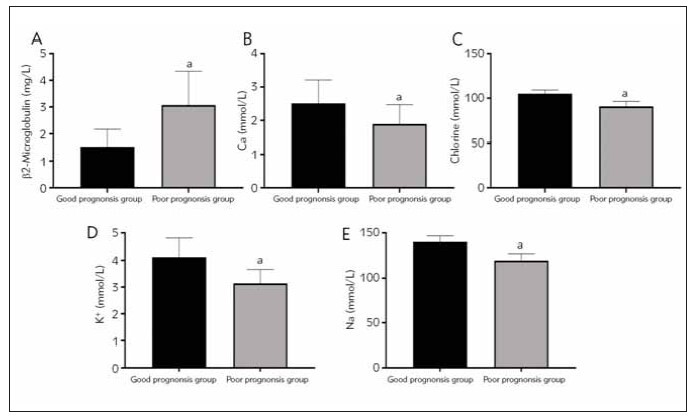
The analysis of β2-microglobulin and electrolyte levels in patients with varying prognoses. (A) comparison of β2-microglobulin levels between the two groups; (B) comparison of calcium levels between the two groups; (C) comparison of chlorine levels between the two groups; (D) comparison of potassium levels between the two groups; (E) comparison of sodium levels between the two groups; Note: aP < 0.05 compared with the good prognosis group.

In contrast to the group with favorable prognosis, the poor prognosis group exhibited a noteworthy elevation in β2-microglobulin levels, accompanied by a significant decrease in calcium, chlorine, potassium, and sodium levels (P < 0.05) according to Table 4 and Figure 2.

**Table 4:** Analysis of β2-microglobulin and electrolyte levels in patients with different prognosis.

Group	N	β2-microglobulin<br>(mg/L)	Calcium<br>(mmol/L)	Chlorine<br>(mmol/L)	Potassium<br>(mmol/L	Sodium<br>(mmol/L)
Good prognosis group	67	1.53±0.64	2.51±0.70	104.93±4.18	4.12±0.69	140.06±6.63
Poor prognosis group	53	3.07±1.27	1.89±0.58	90.71±5.52	3.14±0.52	119.75±7.68
*t*		8.641	5.190	16.060	8.587	15.535
*P*		<0.001	<0.001	<0.001	<0.001	<0.001

### ROC curve analysis

**Figure 3:**
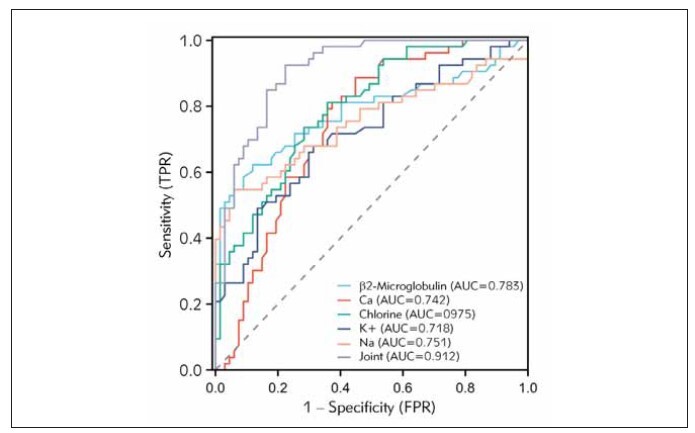
ROC curve analysis of β2-microglobulin and electrolyte levels in predicting the prognosis of COPD.

ROC curve analysis demonstrated that the β2-microglobulin, calcium, and chlorine exhibited an area under the curve of 0.783, with sensitivity and specificity reaching 62.26% and 88.06% respectively. The area under the curve for chlorine detection was calculated as 0.795, while the sensitivity and specificity were determined to be 81.13% and 64.18% respectively. Potassium detection displayed an area under the curve of 0.718, with sensitivity and specificity values of 67.93% and 68.66% respectively. In terms of sodium detection, the area under the curve was found to be 0.751, sensitivity reached 54.72%, and specificity was determined to be 94.03%. Moreover, joint detection exhibited a considerably higher area under the curve of 0.912, as well as a sensitivity and specificity of 92.45% and 77.61% respectively. It is noteworthy that combined detection yielded significantly higher values for the area under the curve, sensitivity, and specificity compared to single detection methods, thus showcasing its potential in predicting the prognosis of COPD. For further details, refer to Table 5 and Figure 3.

**Table 5:** The prognostic value of β2-microglobulin and electrolyte levels in patients with COPD.

Index	Area under curve	Sensitivity (%)	Specificity (%)	Cut-off value	Truncation value	95%CI
β2-microglobulin	0.783	62.26	88.06	36.73	1.86 mg/L	0.694~0.873
Calcium	0.742	88.68	55.22	56.55	2.23 mmol/L	0.654~0.831
Chlorine	0.795	81.13	64.18	44.10	100.59 mmol/L	0.717~0.873
Potassium	0.718	67.93	68.66	54.90	3.97 mmol/L	0.625~0.811
Sodium	0.751	54.72	94.03	48.51	135.88 mmol/L	0.657~0.846
Joint	0.912	92.45	77.61	88.68	—	0.862~0.961

## Discussion

COPD is a prevalent, manageable, and preventable respiratory condition encountered in clinical settings. The primary pathophysiological changes associated with COPD involve limited airflow and airway blockage. Prolonged smoking is currently recognized as the most prevalent and prominent cause, with the additional risk of COPD elevated by prolonged exposure to occupational dust and chemical gases. The deterioration of COPD is a pivotal occurrence in the disease’s progression, potentially leading to increased hospitalization rates and readmissions, as well as exacerbating the condition [10]. It is commonly acknowledged that age, heart failure, and long-term oxygen therapy are influential factors impacting patient prognosis [11] [12]. However, the extent to which β2-microglobulin and electrolyte levels can serve as prognostic indicators necessitates further investigation.

β2-microglobulin, a protein synthesized by various amino acids, plays a crucial role in the immune system. Past research has indicated that it may serve as a significant risk factor for cardiovascular disease and a valuable marker for renal injury. Chronic obstructive pulmonary disease (COPD), a multifaceted pulmonary and systemic condition, involves mechanisms such as inflammation, autophagy, aging, and epithelial-to-mesenchymal transition (EMT) and fibrosis [13] [14]. Moderate to severe COPD patients often exhibit pulmonary diffusion impairment, characterized by EMT of alveolar epithelial cells, thickening of alveolar walls/septa, and damage to the alveolar capillary membrane detected through pulmonary function tests (DLCO and/or DLCO/VA) [15] [16]. Recent findings suggest an increase in the expression of β2-microglobulin in alveolar epithelial cells, indicating its possible involvement in COPD progression. Multiple studies have demonstrated significantly elevated levels of β2-microglobulin in the plasma and alveolar fluid of COPD patients, potentially inducing epithelial cell senescence and contributing to the development of lung diseases. Further investigations have shown that increased concentrations of β2-microglobulin are associated with a higher risk of mortality, rendering it a valuable prognostic indicator for poor outcomes in COPD patients [17] [18] [19] [20] [21]. In the present study, β2-microglobulin levels exhibited a gradual increase corresponding to COPD severity. Logistic regression analysis and Kendall’s tau-b correlation analysis both revealed a significant positive correlation between β2-microglobulin levels and disease severity. Additionally, β2-microglobulin showed promising predictive value in terms of COPD prognosis. These findings collectively underscore the importance of monitoring β2-microglobulin levels, enabling timely evaluation of patient conditions and facilitating early intervention and treatment for COPD, thereby potentially improving patient prognoses. Pulmonary manifestations, such as airway obstruction, alveolar inflammation, and fibrosis in individuals with COPD, contribute to increased permeability of the alveolarcapillary membrane. This heightened permeability leads to the exudation of electrolytes from blood vessels into the alveoli, causing electrolyte imbalances.

Maintaining an appropriate balance of electrolytes is crucial for regulating bodily functions and promoting overall health. Even the slightest deviation from normal levels of electrolyte concentration can lead to a range of issues, including the potential for fatal consequences. However, electrolyte disorders often go unnoticed, particularly in patients with chronic obstructive pulmonary disease (COPD), who may already suffer from complications such as respiratory failure and malnutrition. The occurrence of electrolyte disturbances in patients with COPD can further exacerbate their condition. The causes of electrolyte imbalance in COPD patients may include factors such as malnutrition, which impairs the body’s ability to absorb and utilize electrolytes, as well as the use of certain medications commonly prescribed for COPD, such as bronchodilators, diuretics, and antibiotics. For instance, diuretics can lead to the depletion of potassium and chloride levels, resulting in hypokalemia and hypochloremia respectively. Additionally, COPD patients are at an increased risk of developing respiratory acidosis due to compromised lung function and impaired ventilation capabilities.

In acidosis, the distribution of electrolytes in the intracellular and extracellular fluids will undergo changes, leading to an imbalance in electrolytes. Hence, accurately and promptly identifying potential danger signals of patients and intervening in a timely manner are crucial to reducing mortality and improving the prognosis in the context of COPD and electrolyte disorders. The most common electrolyte disorders involve imbalances in sodium, potassium, chlorine, and calcium. A study on lung disease discovered that deceased patients had significantly lower levels of serum sodium and calcium compared to surviving patients, and these levels showed a negative correlation with the severity of the patients’ condition, potentially serving as protective factors against death [22]. The study’s findings demonstrated that as the disease’s severity increased, the levels of serum calcium, chlorine, potassium, and sodium significantly decreased. Furthermore, poor prognosis patients exhibited lower levels of these electrolytes, emphasizing the significant correlation between serum electrolyte levels and the patients’ condition. Moreover, the study revealed that combined examinations play a vital role in predicting patients’ prognosis. Analysis of electrolyte imbalances in serum may exacerbate respiratory muscle fatigue in COPD patients, as these imbalances affect the ion concentrations in intracellular and extracellular fluids, thereby impacting muscle contraction and relaxation, which aggravates respiratory muscle fatigue [23] [24]. Additionally, electrolyte disturbances can lead to disruptions in body fluid metabolism, contributing to increased pulmonary circulation load and worsening pulmonary hypertension. Furthermore, electrolyte disorders can influence the function of ion channels in cardiomyocytes, resulting in abnormal myocardial contraction and relaxation, thereby elevating the risk of cardiovascular disease [25] [26].

In summary, patients with a more severe form of the disease exhibit elevated levels of β2-microglobulin and decreased levels of electrolytes. The combined assessment of β2-microglobulin and electrolyte levels demonstrates significant prognostic value for patient outcomes. By accurately evaluating β2-microglobulin and electrolyte levels, clinicians can make informed judgments regarding patient conditions and prognosis, enabling the implementation of effective treatment strategies to mitigate disease progression and improve patient prognosis. Nonetheless, it is important to acknowledge the limitations of this study. The sample size of patients included in our study was small, which may have skewed our findings. More prospective multicenter studies are needed to further validate our conclusions. Due to time constraints, only a single serum sample was collected, potentially failing to capture dynamic changes over time. Additionally, despite adjustments for various confounding factors, the possibility of uncontrolled confounding cannot be entirely ruled out. Therefore, future research is warranted to validate these findings.

## Dodatak

### Conflict of interest statement

All the authors declare that they have no conflict of interest in this work.
